# A larger study of food-related IgG confirms the possible new epidemiological approach to non-IgE-mediated reactions and suggests five great food clusters

**DOI:** 10.1186/2045-7022-5-S3-P39

**Published:** 2015-03-30

**Authors:** Francesco Attilio Speciani, Gabriele Piuri, Jacopo Soriano, Enrico Ferrazzi

**Affiliations:** 1 GEK srl, Milan, Italy; 2 SMA srl, Milan, Italy; 3 Duke University, Durham, NC, USA; 4 Department of Woman, Mother and Neonate, University of Milan, Milan, Italy

## Background

Studies in mouse models show 2 pathways of systemic anaphylaxis: the classic one mediated by IgE, FcεRI, mast cells and histamine and platelet-activating factor (PAF) and the alternative way mediated by total IgG, FcγRIII, macrophages, and PAF. The importance of the latter in humans is still uncertain, but human IgG, IgG receptors, macrophages, mediators and their receptors have appropriate properties to support this pathway if enough IgG and antigens are present.[1] Food specific IgG values might reflect the exposure to food clusters in individual diet habits.[2] This suggests new perspectives in understanding non-IgE-related reactions. The aim of this perspective study was to assess the robustness of food antigen cluster analysis[3] on a larger cohort of subjects.

## Methods

Specific total IgG antibodies against 44 common food antigens were measured in sera of 11488 Italian patients (76.1% females; age 46.7 ± 14.2 years). We used an unsupervised hierarchical clustering algorithm to explore varying degrees of similarity among food antigens. The algorithm initially has 44 clusters (one for each antigen) and then gradually groups them together according to their similarities. The algorithm stops when all food antigens belong to the same cluster.[4]

## Results

Five great food clusters have been identified. The first one consists of highly Nickel-containing foods such as tomato, kiwi fruit, peanut, almond and buckwheat. Within this group it is possible to identify the second cluster including wheat and related grains such as kamut, spelt and barley. The third cluster contains dairy products (cow and goat milk as well as parmesan, mozzarella and ricotta cheese). The fourth one includes yeasts such as *Candida albicans* and *Saccharomyces cerevisiae* and contains also porcini and champignon mushrooms. This cluster is probably connected with fermented foods. The last cluster, less detailed, probably contains foods with a higher concentration of natural salicylates: products such as honey, tea, courgettes, oranges and so on.

## Conclusion

These larger data allow an innovative nutritional approach to the diagnosis and therapy of non-IgE-mediated food reactivities. This provides immunological evidence to food cluster nutritional approach to individual patients affected by food hypersensitivities.
